# The Impact of Socioeconomic Factors on Mental Health: A Conceptual Framework

**DOI:** 10.7759/cureus.88244

**Published:** 2025-07-18

**Authors:** Mohamed Salem, James Robenson

**Affiliations:** 1 Biomedical Sciences, Penn State Health Milton S. Hershey Medical Center, Hershey, USA; 2 Cardiothoracic Surgery, AdventHealth Orlando, Orlando, USA

**Keywords:** education level, health disparities, income inequality, intersectionality, mental health, neighborhood environment, psychological well-being, social determinants, social support, socioeconomic status

## Abstract

Income, education, occupation, and social position are all examples of socioeconomic factors that shape mental health outcomes significantly. This review carries a conceptual framework to describe the complex interaction between mental health and these socioeconomic determinants. It integrates existing theoretical models and empirical evidence while highlighting the pathways through which socioeconomic disparities affect psychological well-being. Access to resources, exposure to stressors, neighborhood environment, and social support networks are key mechanisms of change. Further, it considers how race, gender, and age intersect with these variables. The overall objective of this review is to offer an inclusive approach that can be used as a basis for policy formulation aimed at addressing mental health disparities and promoting resilience among different population groups. It highlights a need for a multi-disciplinary approach to political interventions that tackles socioeconomic origins of mental health issues by indicating the requirement for system transformation leading to fairer conditions for persons with mental illness, since it is the only solution that can address the behaviors of individuals in any society equally, regardless of their economic or social background.

## Introduction and background

Mental health is shaped by many factors and is the most vital part of overall health. If we want to understand the mental health situation of one person or a community, we have to consider socioeconomic factors. Socioeconomic status (SES) includes income levels, educational background, types of occupation, as well as social class, which all together determine general living standards and resource availability [1]. These components also influence different aspects of psychological well-being, thus guiding the development of effective intervention strategies and policies aimed at reducing disparities in mental health among various population groups.

Individuals from lower social classes often experience chronic anxiety due to persistent financial instability, unsafe living conditions, or job insecurity. For example, a parent working multiple low-wage jobs may constantly worry about meeting basic needs like rent or food, leading to long-term stress. Over time, this heightened anxiety increases vulnerability to mental health conditions such as depression [1,2]. Additionally, children growing up in deprived households often face multiple adverse childhood experiences (ACEs), including neglect, exposure to domestic violence, or chaotic home environments. These experiences disrupt emotional development and are strongly associated with poor mental health outcomes in adolescence and adulthood [3,4]. Also, SES affects mental health through its influence on social support systems. Good friendships can offer emotional support, practical help, and a sense of belongingness, thus preventing these disorders. However, it can also be argued that individuals from low-income groups often have limited social connections, as they tend to live on the margins of society. This isolation may stem from physical mobility challenges, which reduce their access to protective social networks and support systems [5].

It should also be noted that one’s geographical location has serious implications on his or her psychological well-being. The connection between mental health and the neighborhood is also very important. People with low SES are likely to live in underprivileged areas where crime rates are high, there is pollution everywhere around them, and no access to parks or gardens. Living in unsafe or unstable environments causes chronic stress, which can lead to hopelessness and insecurity, increasing the risk of mental illness. For instance, constant exposure to violence or housing instability can trigger anxiety and depression [1,2]. Besides this, intersectionality makes things even more complex when it comes to understanding how poverty impacts mental health. Intersectional approach takes into account various factors like race, gender, and age, among others, that combined with income level, create different vulnerabilities for different people [4]. For instance, racial minorities face systemic discrimination coupled with limited opportunities for economic growth, so their problems become worse than those of other groups living within similar conditions of lower income levels [3]. Similarly, women may face unique challenges such as gender-based violence, caregiving burdens, or income inequality, which not only worsen mental health early in life but also compound effects in older age [1,2]. 

In view of these intricacies, we need a more comprehensive framework that will help us better understand the relationship between socioeconomic factors and psychological well-being [4]. This model should look at both theoretical perspectives as well as empirical findings so that it can map out some key points through which such linkages occur. It is only by doing this that we shall be able to come up with strategies meant for policy change aimed at addressing disparities in mental health among diverse populations while promoting resilience within them.

This review seeks to provide an overview of the relationship between SES and mental health outcomes indicators. The intention behind it is not just showing how one affects another, but also showing why such a thing happens in the first place. Therefore, it tries to explain all possible means through which poverty can have an effect on mental health. It does so by considering intersections among different variables involved, thus giving a broader reflection about this issue as a whole. In addition, the main aim here is to bring together various disciplines concerned with these issues since they deal with systems.

## Review

Income inequality and adult mental health

Mental illnesses are major drivers of the global burden of disease and are a leading cause of years lived with disability (YLDs) worldwide, according to a review by Tibber et al. [1]. Therefore, there is an escalating call for more investments in psychological therapies within mental health services; nevertheless, this approach largely ignores broader socioeconomic contexts related to mental illness and, therefore, diverts attention from necessary social and economic reforms. The relationship between income and health has been clearly established. For instance, life expectancy tends to increase as gross national product (GNP) rises, but only to a certain extent, where other factors come into play such that further advances can no longer be made [1]. Similarly, mental health follows a similar pattern in which wealthier countries generally have better mental health outcomes. The Income Inequality Hypothesis (IIH) posits that health outcomes do not depend solely on an individual’s own position within society, but also on how much better or worse off he/she is relative to others [6]. Indicators such as the Gini coefficient, which measures income inequality within a population; higher values indicate greater disparity in earnings, reflecting how uneven or "unfair" income distribution is [1]. This concept was introduced by Wilkinson and Pickett through various evidences that indicate higher Gini coefficients are strongly associated with deterioration of overall physical and mental health conditions of global societies [7]. Although several criticisms have been raised against Wilkinson and colleagues' analyses, the principal finding of a positive association between higher inequality and poorer physical health and social outcomes has been confirmed in subsequent studies [1].

One of the explanations regarding how income inequality affects health is provided by three key theories: the Social Capital Hypothesis (SCH) [8], the Status Anxiety Hypothesis (SAH) [9], and the Neo-Materialist Hypothesis (NMH) [10]. People living in close proximity may experience increased stress levels if socioeconomic disparities lead to reductions in trust among them, reducing empathy among individuals who lack the necessary emotional support from friends, which can aggravate their mental wellness [1]. On the other hand, SAH proposes that individuals experiencing comparison with others they perceive as being more advantaged than they are will end up feeling an inferiority complex or mental strain, hence poor health. NMH contends that disparities arising from wage differences due to workplace inequalities between social classes in different industries or sectors create much variation in citizens' disposable incomes. Consequently, reduced government investment in public goods such as healthcare services in highly unequal societies can result in poorer physical health outcomes for the population [1].

The aim of Tibber et al.’s review was to determine how income inequality at the sub-national level affects mental health among adults. They scrutinized data from almost eight million respondents within 110,000 geographical units [1]. It appeared that in 54.76% of all included studies, the IIH was supported; thus, these studies showed that higher income disparities led to poorer mental well-being. On the other hand, 11.9% of these studies proved the Mixed Neighborhood Hypothesis (MNH), which means that sometimes high levels of inequality can lead to better psychological outcomes by merging low-income individuals with their richer neighbors, who may offer more social contacts and economic opportunities [1]. Their review presented a number of methodological deficiencies that appeared in the previous literature on this topic; however, it was noted that most failed to perform multi-level analyses with appropriate control for relevant confounders such as absolute poverty. There was also a lack of robust statistical models and analysis across geographic scales to accurately assess the impact of income disparity on mental health [1]. Additionally, the social drift theory suggests that severe mental illness can lead to downward social mobility, further worsening economic and mental health outcomes [11]. Nevertheless, area-based findings from Tibber et al.’s reviews suggest that, irrespective of other factors, lower community wealth or status is associated with poorer individual well-being [1]. Therefore, policymakers should not just focus their interventions on individuals living in deprived areas but also work towards ensuring fair access to resources across communities, which promotes social capital whilst reducing status anxiety among residents in whole areas.

**Figure 1:**
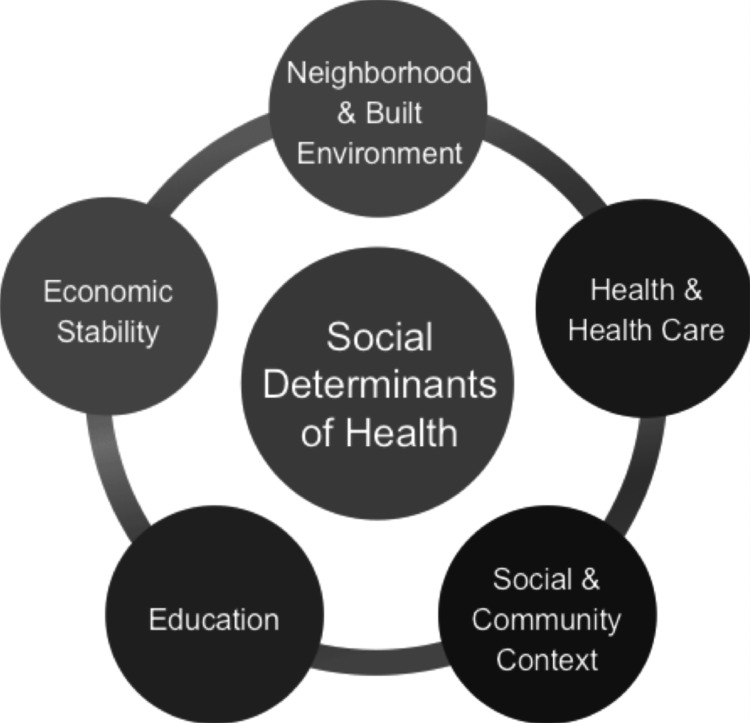
Social determinants of health Image source: Ugwu et al., 2025 [12]; CC BY-NC 4.0, Attribution-NonCommercial 4.0 International

Figure 1 shows the social determinants related to health.

SES and mental health in children and adolescents

According to Reiss et al., mental disorders are a significant contributor to global disease burdens and the number one cause for years lived with disability worldwide [2]. Likewise, children and adolescents from low SES backgrounds have higher rates of mental health problems than their peers from higher SES groups. Their study, the German BELLA cohort study, which examines direct and interactive associations between SES indicators, stressful life events, and mental health problems among children and adolescents, revealed that young people in poverty suffer disproportionately greater levels of mental ill health than their wealthier counterparts. Household income is an indicator of this, as well as parental education level; both lower household incomes and less educated parents were linked with more frequent occurrences of mental disorder amongst children. According to Reiss et al., individuals from lower socioeconomic backgrounds in Germany experienced a higher burden of psychiatric symptoms. However, this cannot be generalized globally.

Reiss et al.'s study also indicated that there is a relationship between tough life situations and severity as well as frequency levels of children’s psychiatric disorders, in case we take into account only families from the very lowest income groups [2]. School-related problems are higher for children from families with severe financial crisis connected to parental illness or unemployment. People living in extreme poverty often experience worse mental health outcomes due to multidimensional deprivation beyond financial hardship [2]. Parental illness, bankruptcy, or loss of employment may have negative impacts on children’s growth, which can be even worse if all these happen amidst limited access/availability of resources for effective coping. This is more frequent within low socio-economic areas and thus, poverty itself leads directly to higher exposure rates for all sorts of risk factors, which may then precipitate the onset of onset of mental ill health conditions [2]. The relationship between SES and mental health is complex, involving various forms of deprivation and exposure to chronic stressors. Parental education was found to be a very strong predictor; children whose parents had the least years of schooling were almost five times more likely to have a psychiatric disorder than those whose parents were better educated. However, there might be some other explanations too, such as genetic vulnerabilities, chronic environmental stress, or limited access to early intervention services that could contribute to the development of psychiatric disorders in children from low SES backgrounds [2].

The study by Reiss et al. has several strengths, but one important limitation: it is not known whether these problems persist over time because only two waves were collected [2]. What is known is that kids who were already showing signs at the baseline assessment point will most likely continue manifesting similar difficulties throughout the next 24-month period. This means that we should try to identify them early on and give continuous help thereafter. Their review further explains that poverty and other financial challenges are connected to poor mental health among children. These findings emphasize the need for policies that aim at bridging the income gap and reducing its negative impact on mental well-being. Such measures may include programmes that provide equal opportunities to resources, foster social integration, as well as decrease status insecurity among people from different backgrounds [2]. In summary, their study shows strong evidence for an association between low SES and exposure to stressful life events with mental ill health in young individuals.

The interplay of SES and stressful life events suggests a more nuanced strategy towards addressing inequities in mental health. This can be achieved by concentrating efforts on diminishing disparities through education support systems for families while also teaching them stress-coping mechanisms; thus, this would contribute greatly to positive psychological outcomes amongst children living in various settings. Additionally, it is important to note that such reviews highlight the strong correlation between poverty levels and mental health across generations, showing that the deeper the poverty, the higher the risk for psychiatric disorders. Families in extreme poverty face chronic stress, inadequate housing, poor nutrition, and limited healthcare access-all of which significantly impact mental well-being. Early adverse life events, such as abuse, neglect, or exposure to violence, are more common in these settings and have been linked to long-term changes in brain development and increased vulnerability to depression, anxiety, and other mental illnesses later in life [2].

**Figure 2:**
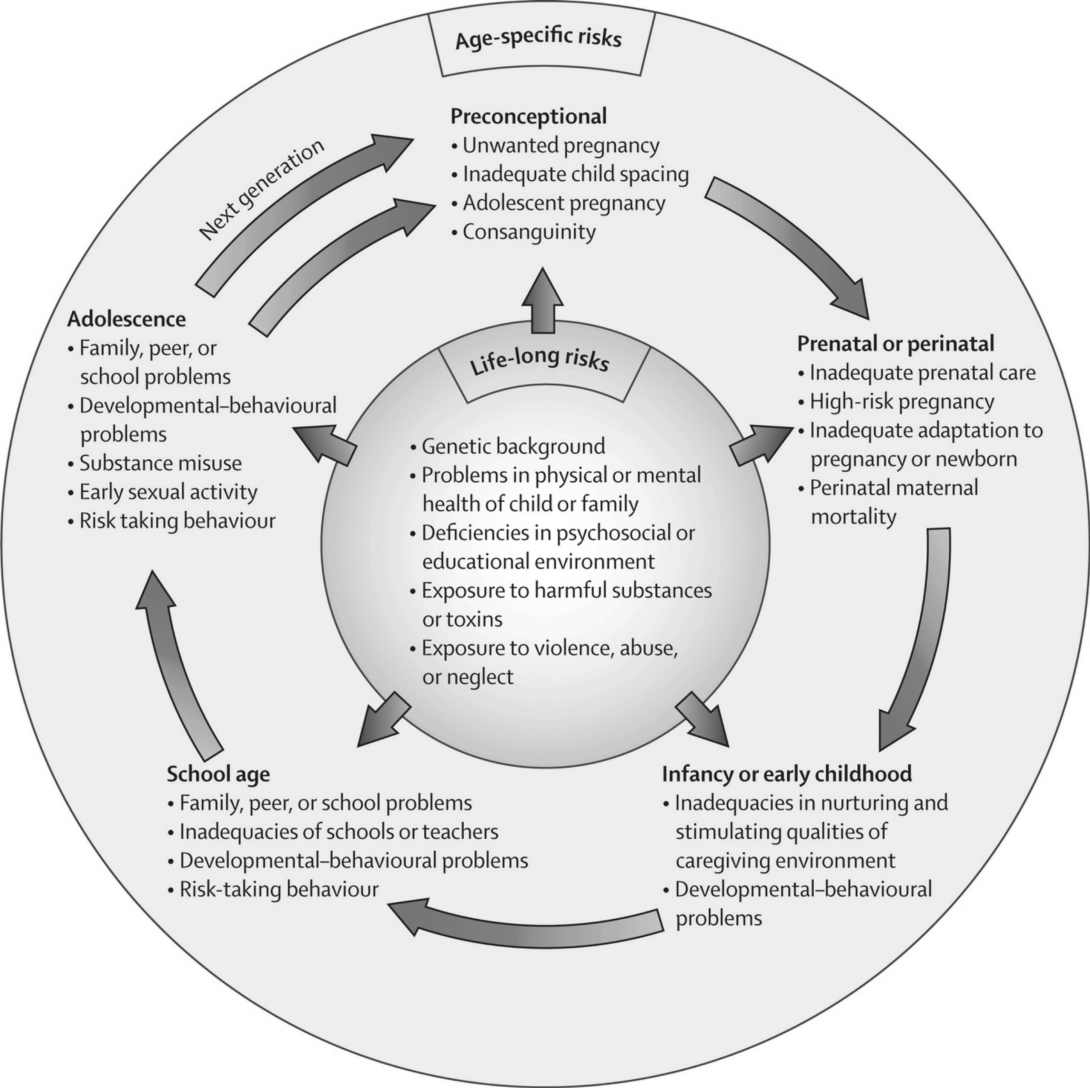
Age-specific and life-long risks for mental health Image Source: Keiling et al., 2011 [13]; used with permission

Figure 2 outlines the various age-specific and life-long risks that can impact mental health, including preconceptional, prenatal, perinatal, infancy, early childhood, school age, and adolescence factors. It highlights how these risks are interconnected and can influence the mental health outcomes of individuals across different stages of life.

Income inequality and depression

Patel et al. conducted a systematic review and meta-analysis indicating that income inequality contributes to poor mental health outcomes [3]. Their meta-analysis also examines depression in relation to income inequality while looking into possible mechanisms. They included 26 studies, mostly conducted in high-income countries, and found that income inequality was positively related to depression risk according to two-thirds of these studies, and five out of six longitudinal studies reported statistically significant positive relationships between them. Only one study found a significant negative relationship. The pooled risk ratio of 12 dichotomized groups representing different levels of inequality was estimated at 1.19, which shows populations living with wider disparities are at higher risks for depressive disorders than those experiencing lesser disparities in wealth distribution within their societies. It implies that there is a 19% increase in odds of having depressive disorder associated with income equality [3].

Several studies pointed out that income inequality affects women and low-income populations more, and these were considered as subgroup effects [14,15]. This review proposes a conceptual model demonstrating how multiple socioeconomic and psychosocial factors interact at the individual, community, and national levels to influence mental health outcomes such as depression and anxiety. According to the NMH, societies with high income inequality tend to underinvest in public goods such as healthcare and education, which in turn negatively affects population health [10]. On the other hand, the SCH argues that communities with eroded trust due to levels of inequality lack the strong bonds necessary for equitable resource sharing, leading to poor mental health as individuals may feel uncared for and lack trust in others [8]. Additionally, according to the SAH, feelings of envy or jealousy arise when individuals compare their possessions with those of others who have more than they do, leading to frustration and ultimately depressive emotions upon realizing the impossibility of attaining things due to perceived unfairness [9]. Psychological stress and social defeat are cited as individual-level mechanisms through which inequality can contribute to depression.

**Figure 3:**
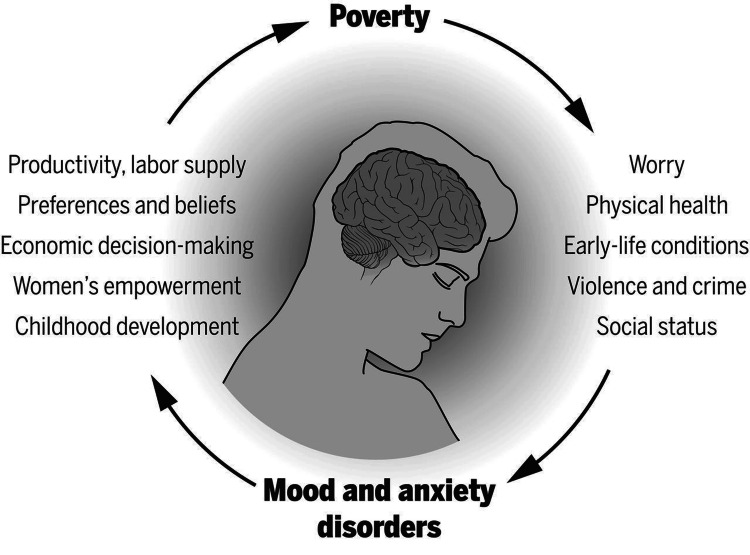
The cycle of poverty and mental health disorders Image Source: Ridley et al., 2020 [16]; used with permission

Figure 3 illustrates the cyclical relationship between poverty and mental health disorders, highlighting how factors such as productivity, economic decision-making, and social status interplay with mood and anxiety disorders, creating a perpetuating cycle. 

The way forward

Policy makers should take up measures that can reduce income disparities, such as progressive taxation and universal basic income. In addition, mental health practitioners must advocate for these policies while advancing interventions targeting pathways leading to depressive disorder through proximal determinants. Adolescent skills development could be enhanced through psychological treatment programs embedded within integrated care bundles. Implementing such programs in low-resource but high-inequality settings poses practical and policy challenges that must be addressed. This review underlines the importance of addressing wealth inequality to improve mental health outcomes across diverse populations.

The strengths of the current review are that it thoroughly combines theoretical models with empirical evidence to form a conceptual framework that demonstrates the intricate link between economic factors and mental health. This involves concepts such as earnings, learning, occupation, and social standing, among other things. Additionally, it highlights intersectionality by spanning race, gender, and age, enabling a very nuanced approach to mental health disparities. Some of the literature reviewed lacked multi-level analysis and failed to control for confounding factors such as absolute poverty, which may have limited the robustness of their findings. The authors of this review recommend the development of interventional policies involving multiple academic disciplines as a strategy to reduce socioeconomic disparities and improve psychological well-being across populations.

## Conclusions

Worldwide, mental illnesses are a significant problem as they remain the most common cause of YLDs. This review demonstrates how income inequality and SES, among other related factors, play a significant role in shaping mental health outcomes across different populations. Also, poor adult mental health is associated with subnational income inequality. As reviewed, communities with significant income disparities often exhibit lower levels of well-being, even when controlling for other variables. This reinforces the need for addressing income inequality in public health planning. Based on this, more needs-based policy interventions are needed to narrow these gaps, which would ensure better public health initiatives aimed at reducing risk factors and promoting resilience to prevent the onset of mental disorders vulnerable socioeconomic groups. This could include increasing access to affordable mental health services, expanding early childhood support programs, and investing in education and employment opportunities for low-income communities.
